# Promotional Language (Hype) in Abstracts of Publications of National Institutes of Health–Funded Research, 1985-2020

**DOI:** 10.1001/jamanetworkopen.2023.48706

**Published:** 2023-12-21

**Authors:** Neil Millar, Bojan Batalo, Brian Budgell

**Affiliations:** 1 University of Tsukuba, Tsukuba, Ibaraki, Japan; 2 National Institute of Advanced Industrial Science and Technology (AIST), Tokyo, Japan; 3 Canadian Memorial Chiropractic College, North York, Ontario, Canada

## Abstract

**Importance:**

Investigators applying for National Institutes of Health (NIH) funding increasingly use promotional language (or hype) that has the potential to undermine objective evaluation. Whether or not the same investigators use hype in subsequent research reports has yet to be investigated.

**Objective:**

To assess changes in the use of hype in journal abstracts reporting research funded by the NIH and to compare those trends with previously reported trends in the associated NIH funding applications.

**Design, Setting, and Participants:**

This cross-sectional study assessed trends (from 1985 to 2020) in the use of promotional adjectives in abstracts of journal articles reporting NIH-funded research, and then compared those trends with previously reported trends for the associated NIH funding applications. Articles included in analyses had abstracts available in PubMed.

**Main Outcomes and Measures:**

Absolute change for the 139 adjective forms that have previously been identified as representing hype in NIH funding applications was measured as the difference in frequency between 1985 and 2020. Relative change was measured as the percentage change in frequency in 2020 relative to 1985, or the first year of occurrence. Consistency of change was measured by the rank order correlation (Kendall τ). Concordance between longitudinal trends in the journal abstracts and NIH funding applications was measured by the rank-order cross-correlation.

**Results:**

In a total of 2 394 480 journal abstracts, all 139 adjective forms were identified in 2 793 592 total occurrences. Among these adjectives, 133 increased in absolute frequency by 5335 words per million (wpm), with a mean (SD) relative increase of 1404% (2371%). The largest absolute increases were for *novel* (524 wpm), *important* (414 wpm), and *key* (378 wpm). The largest relative increases were for *scalable* (22 wpm [19 964%]), *unmet* (23 wpm [12 126%]), and *tailored* (40 wpm [8169%]). The mean (SD) correlation for all adjectives was 0.70 (0.30) with 95 adjectives showing a strong positive correlation (τ > 0.7; *P* < .001), 24 a moderate positive correlation (0.5 < τ < 0.7; *P* < .001), and 3 a moderate negative correlation (−0.5 < τ < −0.7; *P* < .001). The mean (SD) cross-correlation was 0.64 (0.19) with 61 of the 139 adjectives showing a strong positive cross-correlations (τ > 0.7; *P* < .001), 53 a moderate positive cross-correlations (0.5 < τ < 0.7; *P* < .001), and 3 a moderate negative cross-correlation (−0.7 < τ < −0.5; *P* < .001).

**Conclusions and Relevance:**

In this analysis of journal abstracts reporting NIH-funded research from 1985 to 2020, levels of promotional language were found to be increasing and trends were closely associated with previously reported trends in the related NIH funding applications. This suggests that increasing levels of salesmanship may in part be a downstream effect of salesmanship infused during earlier stages of the research cascade.

## Key Points

**Question:**

Is the increasing use of promotional language (often referred to as hype) in National Institutes of Health (NIH) funding applications associated with a similar shift in journal abstracts reporting the results of NIH-funded research?

**Findings:**

This cross-sectional study of 2 394 480 journal abstracts reporting the results of NIH-funded research from 1985 to 2020 found that the use of 133 out of 139 hype adjectives increased and that these trends were positively correlated with previously reported trends in related funding applications.

**Meaning:**

These results suggest that increasing salesmanship in the reporting of research is in part a downstream effect of language choices made during the stage of funding application.

## Teaser

This cross-sectional study of medical journal article abstracts from studies funded by the National Institutes of Health examines trends in the use of hype adjectives to promote the importance of study findings.

## Introduction

As stated by the Consolidated Standards of Reporting Trials (CONSORT) group and pertaining to randomized clinical trials (RCTs), journal abstracts should allow for objective evaluation of an article’s contents: “Clear, transparent, and sufficiently detailed abstracts of conferences and journal articles related to randomized controlled trials are important, because readers often base their assessment of a trial solely on information in the abstract.”^1^ At the same time, and beyond just clinical trials, within an attention economy where increasing numbers of abstracts vie for potential readers, investigators may feel justified in using a modicum of salesmanship to gain advantage. Hence, some tension may exist between the idealized role vs a perceived promotional role of journal article abstracts.

Additionally, in pragmatic terms, prior to the writing of research reports, most investigators have already invested effort in expressing essentially the same thoughts in successful grant applications. It has, for example, been estimated that biomedical researchers spend 20 to 34 days drafting and editing each grant application.^2^ When they come to write subsequent research reports, investigators may, therefore, find it convenient (indeed, necessary) to use actual wordings, promotional or otherwise, from successful grant applications as templates.

A 2022 study^3^ of successful applications for National Institutes of Health (NIH) funding found that from 1985 to 2020 applicants’ use of promotional language (hype) increased substantially. Focusing on adjectives, as they are the word class most associated with evaluation^4^ and can be reliably identified automatically,^5^ the authors identified 139 adjectives that carry a promotional sense, of which 130 increased by a mean (SD) 1378% (3132%). The study found that applicants increasingly promoted the significance (eg, *imperative*, *paramount*), novelty (*revolutionary, ground-breaking*), scale (*massive, vast*) and rigor (*careful, sophisticated*) of the project, the utility of the expected outcomes (*impactful, seamless*), the qualities of the investigators and research environment (*renowned, stellar*), their attitudes (*incredible, exciting*), and the gravity of the problem (*dire, devastating*).

A related analysis^6^ found that the same hype terms were almost all (138 out of 139) used in the NIH’s own funding opportunity announcements, and that trends in their use were correlated with those in grant applications, suggesting that grant applicants’ increased use of hype language may, in part, be a response to instructions from the NIH. However, whether language choices made at the stage of grant applications have a downstream effect on the tone of research reports remains unclear.

This study, therefore, assessed the use of hype in PubMed abstracts resulting from NIH funding and compares those trends with trends previously reported in the related grant applications. Given the importance of NIH funding mechanisms, it was hypothesized that the trends in the use of hype terms in reports of NIH-funded research would mirror those in funding applications.

## Methods

### Study Design

We analyzed published journal abstracts describing research resulting from NIH projects funded from 1985 to 2020, searching for adjectives that have been found to serve as hype in grant applications.^3^ We assessed (1) trends over time in the use of hype, and (2) the association between these trends and those previously found in NIH funding applications.^3^ The study was designed to comply with relevant items of the Strengthening the Reporting of Observational Studies in Epidemiology (STROBE) reporting guidelines for cross-sectional studies. Because the study did not involve human participants, ethical approval and informed consent were waived by the University of Tsukuba institutional review board.

### Identification of Hype Adjectives

The identification of adjectives that serve as hype in grant applications has been described elsewhere.^3^ Here, we provide a brief summary. From all unique funding application abstracts in the NIH archive (901 717 in total), adjectives were automatically extracted and their frequencies in 2020 assessed relative to the earliest available year (1985), and those adjectives that had shifted significantly in frequency (*P* < .05) were identified. Through reading in their sentential context, retained adjectives were classified as hype if over 30% of the usage in a sample was judged to be promotional by both of 2 investigators working independently. The criterion for judging an adjective as promotional within a given sentence was that it could be removed or replaced with a more objective or neutral alternative without altering the information within the sentences (eg, “As such, development of *novel* radiosensitizing agents is of *crucial* importance…”). In total 139 adjectives were identified.

### Data Preprocessing

From the NIH ExPORTER system,^7^ we extracted the unique PubMed identifiers (PMIDs) associated with NIH projects funded from 1985 to 2020. From the PubMed database, using the Entrez Programming Utilities API,^8^ we downloaded all abstracts describing research publications associated with the NIH projects. We compiled the PubMed abstracts as a text corpus, loaded it into the CQPweb corpus analysis system,^9^ and searched it for the 139 hype adjectives identified previously in successful grant applications.^3^

### Statistical Analysis

To enable comparison, we normalized frequency counts for each year to a common base—words per million (wpm). To measure overall change, we calculated (1) the absolute change, which is the difference in normalized frequencies between 1985 and 2020, and (2) the relative change, which is the percentage change in normalized frequency in 2020 compared with 1985, or the first year of occurrence—not all hype adjectives existed in research current in 1985. To measure the consistency of change, following established methodology,^10^ we calculated the rank order correlation (Kendall τ) with statistical significance set at *P* < .001.

To compare longitudinal trends in PubMed abstracts with those found in grant applications, we inspected superimposed plots of the 2 time-series and conducted rank order cross-correlation analysis with a lag of 0 years with statistical significance set at *P* < .001. All statistical tests were 2-sided, and analyses were performed in the R statistical programming environment, version 4.1.0 (R Group for Statistical Computing).

## Results

The resulting corpus, which comprised 2 394 480 journal abstracts (462.2 million words), contained all 139 hype adjectives (total occurrences 2 793 592). In the PubMed abstracts, the overall use of the hype adjectives has increased over time. Among the 139 adjectives, 133 increased cumulatively in absolute frequency by 5335 wpm, with a mean (SD) relative increase of 1404% (2371%). In contrast, only 6 hype adjectives decreased in frequency by 137 wpm and a mean (SD) relative decrease of 12% (11%). The largest absolute increases were for *novel* (524 wpm [803%]), *important* (414 wpm [139%]), *key* (378 wpm [1327%]), *critical* (376 wpm [546%]), and *diverse* (189 wpm [954%]). The largest relative increases were for *scalable* (19 964% [22 wpm]), *unmet* (12 126% [23 wpm]), *tailored* (8169% [40 wpm]), *nuanced* (6700% [6.8 wpm]), and *foundational* (6367% [7.8 wpm]). Statistically significant correlations (*P* < .001) between adjective frequency and year were observed for 132 of the 139 hype adjectives. The mean (SD) correlation coefficient for all adjectives was 0.70 (0.30) with 95 adjectives showing a strong positive correlation (τ > 0.7; *P* < .001), 24 a moderate positive correlation (0.5 < τ < 0.7; *P* < .001), and 3 a moderate negative correlation (−0.5 < τ < −0.7; *P* < .001). The strongest correlations were observed for *diverse* (τ = 0.99; *P* < .001), *key* (τ = 0.98; *P* < .001), *emerging* (τ = 0.97; *P* < .001), *robust* (τ = 0.97; *P* < .001), and *fundamental* (τ = 0.97; *P* < .001). Full results for all adjectives are provided in eTable in Supplement 1.

**Figure 1.**
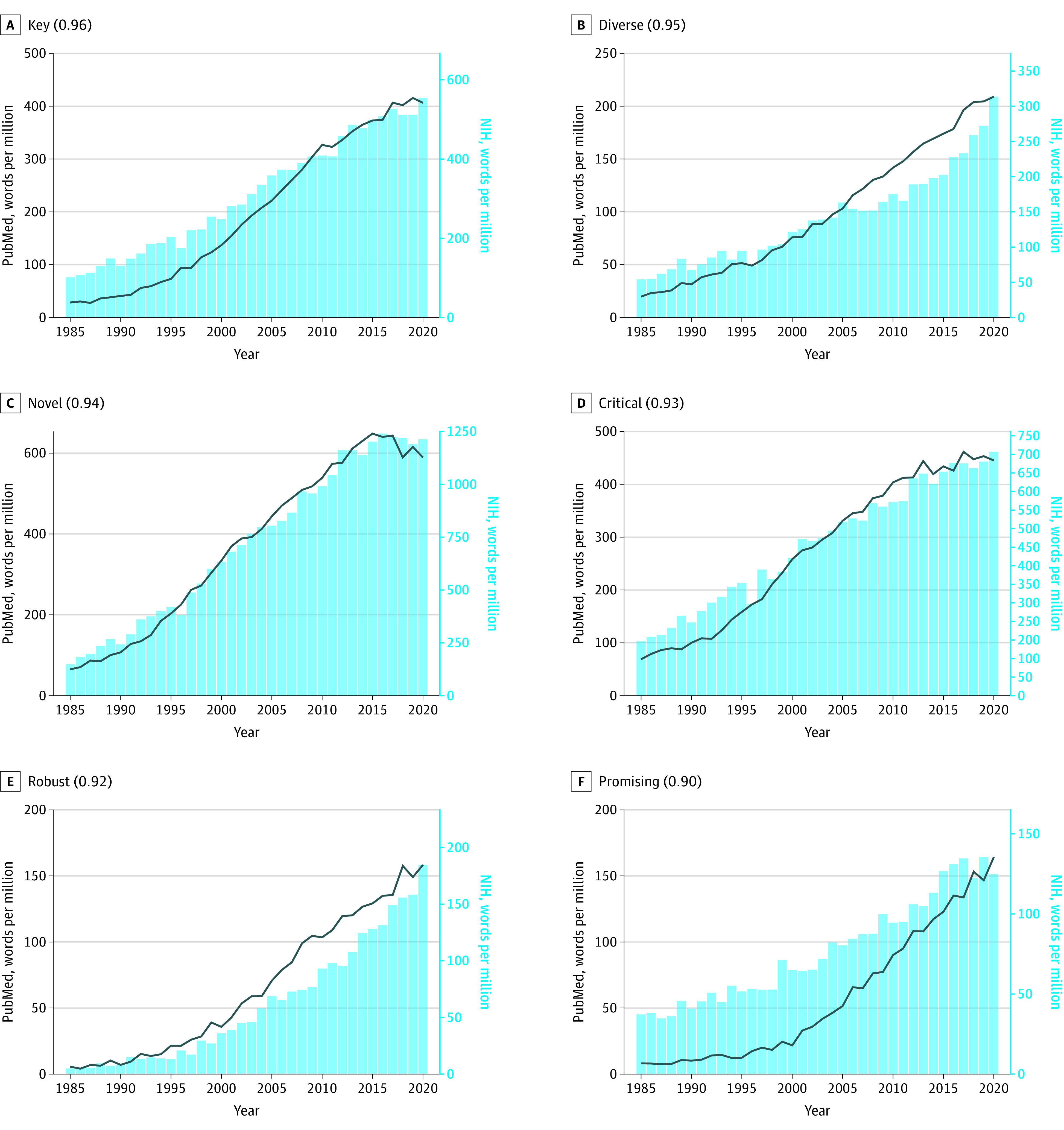
Yearly Frequency of the 6 Most Strongly Cross-Correlated Hype Adjectives in PubMed Abstracts and in National Institutes of Health (NIH) Funding Application Abstracts Frequency of each word’s use in PubMed abstracts and NIH funding application abstracts is shown. In the panel label in parentheses the correlation coefficient between word frequency in the 2 data sets is given.

**Figure 2.**
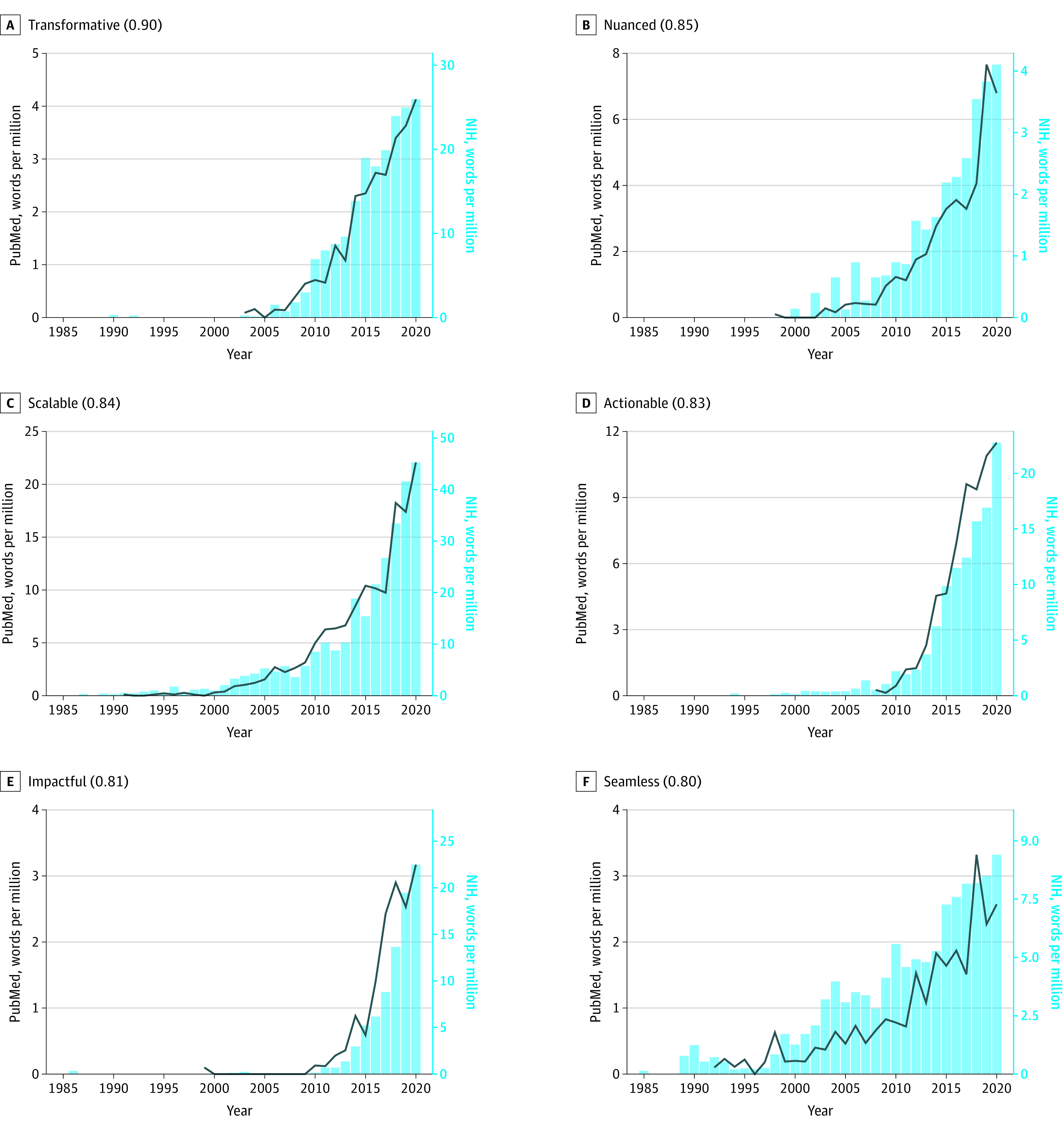
Yearly Frequency of the 6 Most Strongly Cross-Correlated Hype Adjectives in PubMed Abstracts and in National Institutes of Health (NIH) Absent From PubMed Abstracts in 1985 Frequency of each word’s use in PubMed abstracts and NIH funding application abstracts is shown. In the panel label in parentheses the correlation coefficient between word frequency in the 2 data sets is given.

The trends in PubMed abstracts (described above) broadly concord with those reported in NIH funding applications.^3^ Statistically significant cross-correlations (*P* < .001) were observed for 128 out of the 139 adjectives. The mean (SD) cross-correlation was 0.64 (0.19) with 61 of the 139 adjectives showing a strong positive cross-correlation (τ > 0.7; *P* < .001), and 53 a moderate positive cross-correlation (0.5 < τ <0.7; *P* < .001) (Table). Only 3 adjectives showed a moderate negative cross-correlation (−0.7 < τ < −0.5; *P* < .001) (*significant*, *greatest*, *detailed*). The 6 most strongly cross-correlated hype terms were *key *(cross-correlation coefficient, 0.96),* diverse* (0.95)*, novel* (0.94)*, critical* (0.93)*, robust* (0.92)*, *and *promising* (0.90) (Figure 1). The 6 most strongly cross-correlated hype terms that were absent from PubMed abstracts but have since gained popularity were *transformative* (0.90), *nuanced* (0.85), *scalable* (0.84), *actionable* (0.83), *impactful* (0.81), and *seamless* (0.80) (Figure 2). Comparable plots for all adjectives are provided in eFigure in Supplement 1.

**Table.** Hype Adjectives With Moderate to Strong Positive Cross-Correlations

Cross-correlation coefficient	Hype adjectives	*P* value
τ > 0.9	key, diverse, novel, critical, robust, promising, transformative	<.001
0.8 < τ < 0.9	emerging, innovative, unprecedented, relevant, urgent, elusive, nuanced, exciting, scalable, scientific, actionable, successful, unparalleled, compelling, comprehensive, crucial, impactful, seamless	<.001
0.7 < τ < 0.8	efficacious, safer, strong, unmet, quality, rigorous, strategic, top, devastating, essential, myriad, unanswered, vast, tailored, tremendous, advanced, meaningful, timely, unique, broad, durable, imperative, indispensable, sustainable, dismal, motivated, powerful, exceptional, alarming, dedicated, surprising, vital, foundational, groundbreaking, ideal, longstanding	<.001
0.6 < τ < 0.7	efficient, huge, outstanding, daunting, paramount, remarkable, vibrant, transdisciplinary, immense, intuitive, dire, pivotal, confident, user-friendly, expansive, generalizable, substantial, enormous, sophisticated, ready, invaluable, multidisciplinary, notable, deeper, fundamental, overwhelming, premier, effective, skilled, accessible, easy, international, interdisciplinary	<.001
0.5 < τ < 0.6	renowned, intriguing, latest, deployable, incredible, interprofessional, intellectual, revolutionary, senior, accurate, qualified, experienced, fastest, prestigious, ambitious, biggest, attractive, massive, immediate	<.001

## Discussion

Our analyses indicate that hype is increasing in PubMed abstracts that describe the outcomes of NIH funded research. The largest increases are among terms that promote novelty (eg, *novel, innovative*) and importance (*crucial*, *key*), and the most rapid increases are among words that promote utility (*transformative, scalable*, *actionable*, *impactful*). Close concordance with previously reported trends in NIH funding applications,^3^ and funding opportunity announcements,^6^ suggests that changes in the word choices in PubMed abstracts may, in part, be a downstream effect of salesmanship infused during the stage of application for funding.

Taken with previous work,^3,6^ this study rounds out an assessment of trends in the use of hype across the NIH research cascade from funding opportunity announcement to the final publication of research findings. Some disagreement may exist concerning classification of certain adjectives as hype. This notwithstanding, our list, which was derived from population level data, bears a similar vocabulary to that used in other studies of promotional language in research.^11,12,13,14^ Thus, taken as a whole, salesmanship appears to be increasing across the NIH system.

This conclusion is supported by related linguistic analysis^15^ of epistemic stance—that is, confidence and certainty toward knowledge and beliefs. During the same period, applicants for NIH funding have adopted a stance that is increasingly less cautious and less tentative, and increasingly confident, optimistic, and promissory. This is evidenced, for example, by a consistent decline among adverbs that convey weak possibility (eg, *possibly*, *probably, perhaps*) vs an increase among verbs that label propositions as objectively verifiable (*demonstrate*, *establish*, *reveal*).

Concerns that hype may bias readers’ evaluation of research have been raised.^16,17,18^ A study of successful and unsuccessful grant applications by European researchers^19^ found that self-confidence and confidence in the project (ie, a strong epistemic stance) were associated with increased likelihood of application success. Whether or not hype in research reports affects evaluation has not yet been demonstrated. Other concerns that have been raised include that hype may impair the ability of science to find true effects,^14^ lead to redundancy and impair clarity,^3^ and/or fuel skepticism and alienate the reader.^13,20,21^

Our assessment is suggestive of the role that NIH funding systems play in the growth of salesmanship within the research enterprise. Grant applications may nudge investigators to justify their research in overtly promotional terms, as might be illustrated by a typical 2020 NIH funding opportunity announcement: “Discuss how you will continue to maintain a trajectory of creative, groundbreaking, paradigm-shifting, or transformative research and to conduct rigorous, reproducible, and transparent research in your proposed research program.”^22^ Thus, alongside increasing use of hype terms in funding opportunity announcements,^6^ it is perhaps not surprising that we observe similar trends in grant applications,^3^ and, as shown here, in subsequent research reports. Other factors have been discussed elsewhere and include an increasingly competitive research environment, changing structural frameworks, broader societal and technological change, and established processes of language change.^3,15,23^

### Limitations

Our study has several limitations. First, some adjectives identified as representing hype in grant applications may be used with a different meaning in the context of PubMed abstracts. For example, although the adjective *significant* was used predominantly as a synonym for *important* in funding applications (and thus was classified as hype), in PubMed abstracts it primarily refers to statistical significance. In addition, our categorization of adjectives as hype involved some interpretation of the writers’ intent, and as such remains subjective. Moreover, our categorization does not capture the gradation in promotional connotation that exists among the terms. For example, the adjectives *novel* and *innovative* are arguably less hyperbolic than *groundbreaking* and *revolutionary*. Finally, our analysis focused only on monotonic trends—ie, patterns of change that consistently move in one direction.

## Conclusions

In this analysis of journal abstracts reporting NIH-funded research from 1985 to 2020, levels of promotional language were found to be increasing and trends were closely associated with previously reported trends in the related NIH funding applications. Various groups participate in and share responsibility for communication of science. These groups include investigators, reviewers, editors, research-performing organizations and funding bodies. Noting that few studies are the first of their kind, transformative or critically important, a 2022 opinion piece^24^ emphasized the responsibility of individual investigators to be circumspect and avoid hype. The present study points to the role that structural frameworks (the NIH funding mechanisms) play in shaping the tone of research communication. Recognizing the potential for hype to undermine the fidelity of research reports, funding bodies, alongside other groups, share a responsibility to not nudge investigators, and thus the collective system, toward hype.

## Notes

Supplement 1. **eTable.** Frequency in and Change of 139 Hype Adjectives Between 1985 and 2020 **eFigure.** Yearly Frequency in PubMed Abstracts and in NIH Funding Application Abstracts of Hype Adjectives in Descending Order of Cross-Correlation

Supplement 2.
Data Sharing Statement
