# Supplementary material for: Promotional Language (Hype) in Abstracts of Publications of National Institutes of Health–Funded Research, 1985-2020
Source: JAMA Netw Open. 2023 Dec 21;6(12):e2348706. doi: 10.1001/jamanetworkopen.2023.48706 (PMC10739072; doi:10.1001/jamanetworkopen.2023.48706)
Supplement: Supplement 1. — eTable. Frequency in and Change of 139 Hype Adjectives Between 1985 and 2020 eFigure. Yearly Frequency in PubMed Abstracts and in NIH Funding Application Abstracts of Hype Adjectives in Descending Order of Cross-Correlation [file jamanetwopen-e2348706-s001.pdf]

Supplemental Online Content

Millar N, Batalo B, Budgell B. Promotional language (hype) in abstracts of publications of National Institutes of Health–funded research, 1985-2020. *JAMA Netw Open.* 2023;6(12):e2348706. doi:10.1001/jamanetworkopen.2023.48706

eTable. Frequency in and Change of 139 Hype Adjectives Between 1985 and 2020

eFigure. Yearly Frequency in PubMed Abstracts and in NIH Funding Application Abstracts of Hype Adjectives in Descending Order of Cross-Correlation

This supplemental material has been provided by the authors to give readers additional information about their work.

© 2023 Millar N et al. *JAMA Network Open.*

eTable. Frequency changes and temporal correlations in PubMed abstracts and NIH abstracts and cross-correlations for 139 hype adjectives

**term**	**PubMed abstracts**				**NIH abstracts**				**PubMed vs. NIH**	
	absolute change	relative change	Correlation τ (p-value)		absolute change	relative change	Correlation τ (p-value)		Cross correlation τ (p-value)	
accessible	24.9 wpm	+173%	0.77	(P < 0.001)	34.9 wpm	+129%	0.72	(P < 0.001)	0.61	(P < 0.001)
accurate	98.6 wpm	+277%	0.86	(P < 0.001)	32.9 wpm	+55%	0.58	(P < 0.001)	0.58	(P < 0.001)
actionable	11.5 wpm	+4319%	0.95	(P < 0.001)	22.7 wpm	+16114%	0.90	(P < 0.001)	0.83	(P < 0.001)
advanced	92.1 wpm	+220%	0.82	(P < 0.001)	183.1 wpm	+454%	0.89	(P < 0.001)	0.75	(P < 0.001)
alarming	3.0 wpm	+788%	0.77	(P < 0.001)	7.0 wpm	+1257%	0.76	(P < 0.001)	0.71	(P < 0.001)
ambitious	1.2 wpm	+625%	0.63	(P < 0.001)	5.0 wpm	+592%	0.81	(P < 0.001)	0.54	(P < 0.001)
ample	2.5 wpm	+257%	0.61	(P < 0.001)	5.7 wpm	+257%	0.57	(P < 0.001)	0.42	(P < 0.001)
attractive	35.6 wpm	+740%	0.88	(P < 0.001)	12.0 wpm	+98%	0.49	(P < 0.001)	0.52	(P < 0.001)
biggest	2.4 wpm	+665%	0.69	(P < 0.001)	4.3 wpm	+1017%	0.63	(P < 0.001)	0.53	(P < 0.001)
broad	92.8 wpm	+200%	0.93	(P < 0.001)	95.4 wpm	+115%	0.78	(P < 0.001)	0.74	(P < 0.001)
careful	2.4 wpm	+21%	0.49	(P < 0.001)	-16.8 wpm	-64%	-0.51	(P < 0.001)	-0.24	(P < 0.05)
cohesive	1.1 wpm	+46%	0.34	(P < 0.01)	5.7 wpm	+216%	0.63	(P < 0.001)	0.31	(P < 0.01)
collegial	0.2 wpm	+36%	-0.07	ns	3.0 wpm	+782%	0.68	(P < 0.001)	0.27	(P < 0.05)
compelling	11.1 wpm	+574%	0.87	(P < 0.001)	25.7 wpm	+528%	0.87	(P < 0.001)	0.81	(P < 0.001)
comprehensive	108 wpm	+1145%	0.96	(P < 0.001)	131.4 wpm	+151%	0.83	(P < 0.001)	0.81	(P < 0.001)
confident	2.3 wpm	+292%	0.68	(P < 0.001)	4.7 wpm	+339%	0.78	(P < 0.001)	0.65	(P < 0.001)
considerable	-2.8 wpm	-5%	0.38	(P < 0.01)	-38.1 wpm	-51%	-0.86	(P < 0.001)	-0.39	(P < 0.001)
creative	4.5 wpm	+2368%	0.68	(P < 0.001)	6.6 wpm	+530%	0.67	(P < 0.001)	0.45	(P < 0.001)
critical	376.3 wpm	+546%	0.97	(P < 0.001)	555.3 wpm	+348%	0.95	(P < 0.001)	0.93	(P < 0.001)
crucial	97.3 wpm	+1123%	0.95	(P < 0.001)	55.4 wpm	+115%	0.82	(P < 0.001)	0.81	(P < 0.001)

© 2023 Millar N et al. *JAMA Network Open.*

daunting	2.8 wpm	+2418%	0.84	(P < 0.001)	3.3 wpm	+1115%	0.71	(P < 0.001)	0.68	(P < 0.001)
dedicated	13.8 wpm	+3531%	0.87	(P < 0.001)	4.6 wpm	+1643%	0.74	(P < 0.001)	0.71	(P < 0.001)
deeper	10.6 wpm	+149%	0.53	(P < 0.001)	21.7 wpm	+332%	0.77	(P < 0.001)	0.63	(P < 0.001)
deployable	1.0 wpm	+860%	0.73	(P < 0.001)	3.0 wpm	+1150%	0.70	(P < 0.001)	0.59	(P < 0.001)
desperate	0.6 wpm	+300%	0.47	(P < 0.001)	2.1 wpm	+565%	0.65	(P < 0.001)	0.48	(P < 0.001)
detailed	33.6 wpm	+82%	0.82	(P < 0.001)	-121.3 wpm	-54%	-0.89	(P < 0.001)	-0.76	(P < 0.001)
devastating	21 wpm	+5379%	0.92	(P < 0.001)	39.5 wpm	+946%	0.83	(P < 0.001)	0.77	(P < 0.001)
dire	1.4 wpm	+1075%	0.72	(P < 0.001)	4.2 wpm	+2979%	0.76	(P < 0.001)	0.66	(P < 0.001)
dismal	5.1 wpm	+2684%	0.8	(P < 0.001)	4.8 wpm	+1707%	0.85	(P < 0.001)	0.73	(P < 0.001)
diverse	189.2 wpm	+954%	0.99	(P < 0.001)	258.6 wpm	+479%	0.94	(P < 0.001)	0.95	(P < 0.001)
durable	14.6 wpm	+2516%	0.78	(P < 0.001)	22.9 wpm	+2725%	0.89	(P < 0.001)	0.74	(P < 0.001)
easy	24.6 wpm	+532%	0.78	(P < 0.001)	23.7 wpm	+254%	0.69	(P < 0.001)	0.61	(P < 0.001)
effective	158.2 wpm	+68%	0.66	(P < 0.001)	314.4 wpm	+135%	0.88	(P < 0.001)	0.62	(P < 0.001)
efficacious	23.3 wpm	+550%	0.9	(P < 0.001)	25.3 wpm	+233%	0.83	(P < 0.001)	0.79	(P < 0.001)
efficient	83.9 wpm	+162%	0.88	(P < 0.001)	63.9 wpm	+97%	0.77	(P < 0.001)	0.69	(P < 0.001)
elusive	38.6 wpm	+2234%	0.96	(P < 0.001)	22.7 wpm	+652%	0.88	(P < 0.001)	0.85	(P < 0.001)
emerging	164.9 wpm	+3428%	0.97	(P < 0.001)	29.6 wpm	+1636%	0.90	(P < 0.001)	0.87	(P < 0.001)
enormous	9.0 wpm	+587%	0.79	(P < 0.001)	18.1 wpm	+309%	0.69	(P < 0.001)	0.64	(P < 0.001)
essential	171.8 wpm	+180%	0.78	(P < 0.001)	92.6 wpm	+47%	0.71	(P < 0.001)	0.77	(P < 0.001)
exceptional	6.3 wpm	+539%	0.7	(P < 0.001)	32.5 wpm	+1372%	0.85	(P < 0.001)	0.72	(P < 0.001)
exciting	17.7 wpm	+916%	0.9	(P < 0.001)	41.6 wpm	+483%	0.86	(P < 0.001)	0.84	(P < 0.001)
expansive	2.9 wpm	+1516%	0.7	(P < 0.001)	2.9 wpm	+2043%	0.74	(P < 0.001)	0.65	(P < 0.001)
experienced	4.8 wpm	+276%	0.62	(P < 0.001)	13.2 wpm	+263%	0.80	(P < 0.001)	0.56	(P < 0.001)
extensive	4.3 wpm	+5%	0.24	(P < 0.05)	43.6 wpm	+41%	0.67	(P < 0.001)	0.30	(P < 0.01)
fastest	2.3 wpm	+85%	0.57	(P < 0.001)	5.7 wpm	+517%	0.77	(P < 0.001)	0.56	(P < 0.05)
first	49.2 wpm	+10%	0.68	(P < 0.001)	68.3 wpm	+15%	0.24	(P < 0.05)	0.28	(P < 0.001)
foundational	7.8 wpm	+6367%	0.81	(P < 0.001)	24.7 wpm	+6226%	0.78	(P < 0.001)	0.70	(P < 0.001)

© 2023 Millar N et al. *JAMA Network Open.*

fundamental	74.7 wpm	+473%	0.97	(P < 0.001)	50.6 wpm	+34%	0.62	(P < 0.001)	0.63	(P < 0.001)
generalizable	8.5 wpm	+4463%	0.82	(P < 0.001)	13.3 wpm	+598%	0.68	(P < 0.001)	0.64	(P < 0.001)
greatest	-14.2 wpm	-32%	-0.64	(P < 0.001)	19.2 wpm	+134%	0.79	(P < 0.001)	-0.55	(P < 0.001)
groundbreaking	1.0 wpm	+621%	0.7	(P < 0.001)	2.3 wpm	+655%	0.80	(P < 0.001)	0.70	(P < 0.001)
huge	4.6 wpm	+2395%	0.8	(P < 0.001)	7.1 wpm	+849%	0.79	(P < 0.001)	0.68	(P < 0.001)
ideal	22.9 wpm	+198%	0.83	(P < 0.001)	37.1 wpm	+149%	0.80	(P < 0.001)	0.70	(P < 0.001)
immediate	-5.6 wpm	-13%	-0.52	(P < 0.001)	-23.5 wpm	-30%	-0.62	(P < 0.001)	0.50	(P < 0.001)
immense	4.1 wpm	+1051%	0.78	(P < 0.001)	5.6 wpm	+500%	0.71	(P < 0.001)	0.66	(P < 0.001)
impactful	3.2 wpm	+2782%	0.77	(P < 0.001)	22.3 wpm	+6465%	0.67	(P < 0.001)	0.81	(P < 0.001)
imperative	13.3 wpm	+2298%	0.89	(P < 0.001)	15.4 wpm	+347%	0.76	(P < 0.001)	0.74	(P < 0.001)
important	414.3 wpm	+139%	0.77	(P < 0.001)	-147.2 wpm	-19%	-0.17	ns	0.04	ns
incredible	1.4 wpm	+700%	0.67	(P < 0.001)	3.4 wpm	+894%	0.66	(P < 0.001)	0.59	(P < 0.001)
indispensable	7.5 wpm	+486%	0.84	(P < 0.001)	6.3 wpm	+453%	0.79	(P < 0.001)	0.74	(P < 0.001)
innovative	34.1 wpm	+2524%	0.92	(P < 0.001)	390.6 wpm	+2048%	0.93	(P < 0.001)	0.87	(P < 0.001)
intellectual	18.6 wpm	+419%	0.62	(P < 0.001)	28.8 wpm	+218%	0.75	(P < 0.001)	0.59	(P < 0.001)
interdisciplinary	10.4 wpm	+773%	0.78	(P < 0.001)	79.3 wpm	+247%	0.70	(P < 0.001)	0.60	(P < 0.001)
interesting	2.3 wpm	+17%	0.46	(P < 0.001)	-22.0 wpm	-73%	-0.80	(P < 0.001)	-0.41	(P < 0.001)
international	55.2 wpm	+1367%	0.92	(P < 0.001)	41.9 wpm	+243%	0.66	(P < 0.001)	0.61	(P < 0.001)
interprofessional	3.8 wpm	+2124%	0.67	(P < 0.001)	3.0 wpm	+868%	0.46	(P < 0.001)	0.59	(P < 0.001)
intriguing	7.1 wpm	+370%	0.71	(P < 0.001)	7.0 wpm	+145%	0.63	(P < 0.001)	0.59	(P < 0.001)
intuitive	8.0 wpm	+2256%	0.87	(P < 0.001)	3.9 wpm	+704%	0.72	(P < 0.001)	0.66	(P < 0.001)
invaluable	4.7 wpm	+270%	0.83	(P < 0.001)	11.4 wpm	+190%	0.64	(P < 0.001)	0.63	(P < 0.001)
key	378.1 wpm	+1327%	0.98	(P < 0.001)	460.7 wpm	+446%	0.97	(P < 0.001)	0.96	(P < 0.001)
largest	15.6 wpm	+64%	0.4	(P < 0.001)	44.5 wpm	+352%	0.87	(P < 0.001)	0.46	(P < 0.001)
latest	13 wpm	+1354%	0.82	(P < 0.001)	18.2 wpm	+397%	0.71	(P < 0.001)	0.59	(P < 0.001)
longstanding	3.7 wpm	+317%	0.8	(P < 0.001)	13.0 wpm	+665%	0.80	(P < 0.001)	0.70	(P < 0.001)
major	-47.7 wpm	-11%	-0.16	ns	-260.9 wpm	-33%	-0.79	(P < 0.001)	0.19	ns

© 2023 Millar N et al. *JAMA Network Open.*

massive	5.4 wpm	+48%	0.54	(P < 0.001)	9.9 wpm	+108%	0.71	(P < 0.001)	0.51	(P < 0.001)
meaningful	29.3 wpm	+526%	0.84	(P < 0.001)	26.3 wpm	+166%	0.77	(P < 0.001)	0.75	(P < 0.001)
motivated	21.2 wpm	+549%	0.84	(P < 0.001)	6.4 wpm	+192%	0.70	(P < 0.001)	0.73	(P < 0.001)
multidisciplinary	19.6 wpm	+1135%	0.85	(P < 0.001)	59.2 wpm	+89%	0.71	(P < 0.001)	0.63	(P < 0.001)
myriad	14.6 wpm	+2516%	0.89	(P < 0.001)	6.2 wpm	+1105%	0.81	(P < 0.001)	0.77	(P < 0.001)
notable	10.6 wpm	+203%	0.76	(P < 0.001)	7.7 wpm	+291%	0.74	(P < 0.001)	0.63	(P < 0.001)
novel	524 wpm	+803%	0.94	(P < 0.001)	1054.4 wpm	+717%	0.95	(P < 0.001)	0.94	(P < 0.001)
nuanced	6.8 wpm	+6700%	0.9	(P < 0.001)	4.0 wpm	+2779%	0.86	(P < 0.001)	0.85	(P < 0.001)
outstanding	6.5 wpm	+1126%	0.84	(P < 0.001)	64.0 wpm	+837%	0.80	(P < 0.001)	0.68	(P < 0.001)
overwhelming	3.2 wpm	+183%	0.62	(P < 0.001)	4.9 wpm	+236%	0.70	(P < 0.001)	0.63	(P < 0.001)
paramount	6.7 wpm	+693%	0.77	(P < 0.001)	6.4 wpm	+287%	0.76	(P < 0.001)	0.67	(P < 0.001)
pivotal	21.4 wpm	+617%	0.83	(P < 0.001)	16.3 wpm	+189%	0.68	(P < 0.001)	0.66	(P < 0.001)
powerful	63.8 wpm	+453%	0.93	(P < 0.001)	44.3 wpm	+101%	0.79	(P < 0.001)	0.73	(P < 0.001)
premier	0.8 wpm	+591%	0.58	(P < 0.001)	5.6 wpm	+3986%	0.75	(P < 0.001)	0.62	(P < 0.001)
prestigious	0.1 wpm	-29%	0.48	(P < 0.001)	2.3 wpm	+562%	0.59	(P < 0.001)	0.55	(P < 0.001)
productive	6.4 wpm	+104%	0.31	(P < 0.01)	28.5 wpm	+155%	0.70	(P < 0.001)	0.22	ns
promising	156.3 wpm	+1931%	0.95	(P < 0.001)	86.7 wpm	+235%	0.90	(P < 0.001)	0.90	(P < 0.001)
qualified	5.1 wpm	+908%	0.72	(P < 0.001)	23.5 wpm	+313%	0.67	(P < 0.001)	0.57	(P < 0.001)
quality	100.2 wpm	+2263%	0.95	(P < 0.001)	72.0 wpm	+347%	0.79	(P < 0.001)	0.78	(P < 0.001)
ready	3.3 wpm	+100%	0.61	(P < 0.001)	23.9 wpm	+323%	0.75	(P < 0.001)	0.64	(P < 0.001)
relevant	172.2 wpm	+422%	0.97	(P < 0.001)	173.0 wpm	+100%	0.87	(P < 0.001)	0.86	(P < 0.001)
remarkable	12.9 wpm	+96%	0.81	(P < 0.001)	10.4 wpm	+93%	0.69	(P < 0.001)	0.67	(P < 0.001)
renowned	0.4 wpm	+186%	0.57	(P < 0.001)	6.4 wpm	+1776%	0.86	(P < 0.001)	0.59	(P < 0.001)
reproducible	3.5 wpm	+15%	0.16	ns	14.0 wpm	+88%	0.02	ns	0.33	(P < 0.05)
revolutionary	1.7 wpm	+1121%	0.61	(P < 0.001)	4.7 wpm	+1129%	0.74	(P < 0.001)	0.59	(P < 0.001)
rich	4.4 wpm	+6%	-0.16	ns	34.2 wpm	+94%	0.81	(P < 0.001)	-0.14	ns
rigorous	19.8 wpm	+641%	0.9	(P < 0.001)	87.8 wpm	+601%	0.86	(P < 0.001)	0.78	(P < 0.001)

© 2023 Millar N et al. *JAMA Network Open.*

robust	152.8 wpm	+2738%	0.97	(P < 0.001)	177.8 wpm	+3652%	0.96	(P < 0.001)	0.92	(P < 0.001)
safer	10.7 wpm	+1392%	0.87	(P < 0.001)	10.3 wpm	+152%	0.76	(P < 0.001)	0.79	(P < 0.001)
scalable	22.1 wpm	+19964%	0.9	(P < 0.001)	44.6 wpm	+13029%	0.90	(P < 0.001)	0.84	(P < 0.001)
scientific	69.6 wpm	+1809%	0.93	(P < 0.001)	333.9 wpm	+413%	0.86	(P < 0.001)	0.84	(P < 0.001)
seamless	2.6 wpm	+2470%	0.81	(P < 0.001)	9.2 wpm	+6600%	0.84	(P < 0.001)	0.80	(P < 0.001)
senior	3.9 wpm	+674%	0.68	(P < 0.001)	38.7 wpm	+435%	0.80	(P < 0.001)	0.58	(P < 0.001)
significant	-66.2 wpm	-9%	-0.54	(P < 0.001)	250.5 wpm	+91%	0.87	(P < 0.001)	-0.51	(P < 0.001)
skilled	7.5 wpm	+557%	0.72	(P < 0.001)	10.0 wpm	+231%	0.75	(P < 0.001)	0.62	(P < 0.001)
sophisticated	13.7 wpm	+1178%	0.83	(P < 0.001)	17.1 wpm	+94%	0.61	(P < 0.001)	0.64	(P < 0.001)
stark	2.2 wpm	+1206%	0.78	(P < 0.001)	2.1 wpm	+122%	0.46	(P < 0.001)	0.43	(P < 0.001)
stellar	0.2 wpm	-48%	0.11	ns	2.3 wpm	+626%	0.65	(P < 0.001)	0.13	ns
strategic	7.8 wpm	+1013%	0.84	(P < 0.001)	53.5 wpm	+1038%	0.88	(P < 0.001)	0.78	(P < 0.001)
strong	96.1 wpm	+91%	0.84	(P < 0.001)	168.5 wpm	+275%	0.92	(P < 0.001)	0.79	(P < 0.001)
substantial	21.7 wpm	+28%	0.72	(P < 0.001)	63.7 wpm	+143%	0.86	(P < 0.001)	0.64	(P < 0.001)
successful	59.5 wpm	+171%	0.85	(P < 0.001)	268.6 wpm	+260%	0.93	(P < 0.001)	0.82	(P < 0.001)
surprising	4.2 wpm	+84%	0.67	(P < 0.001)	6.3 wpm	+151%	0.72	(P < 0.001)	0.71	(P < 0.001)
sustainable	11.1 wpm	+2833%	0.74	(P < 0.001)	35.2 wpm	+25157%	0.92	(P < 0.001)	0.74	(P < 0.001)
synergistic	1.4 wpm	+6%	-0.17	ns	24.4 wpm	+143%	0.72	(P < 0.001)	-0.12	ns
systematic	70.2 wpm	+486%	0.9	(P < 0.001)	-19.3 wpm	-28%	-0.28	(P < 0.05)	-0.23	(P < 0.05)
tailored	31.9 wpm	+8169%	0.93	(P < 0.001)	5.9 wpm	+4186%	0.80	(P < 0.001)	0.76	(P < 0.001)
talented	0.3 wpm	+88%	0.43	(P < 0.001)	11.4 wpm	+1629%	0.78	(P < 0.001)	0.47	(P < 0.001)
tangible	1.8 wpm	+963%	0.55	(P < 0.001)	3.5 wpm	+838%	0.76	(P < 0.001)	0.42	(P < 0.001)
timely	23.1 wpm	+2404%	0.89	(P < 0.001)	50.0 wpm	+561%	0.82	(P < 0.001)	0.75	(P < 0.001)
top	44.4 wpm	+536%	0.86	(P < 0.001)	42.5 wpm	+1221%	0.86	(P < 0.001)	0.78	(P < 0.001)
transdisciplinary	2.8 wpm	+1285%	0.59	(P < 0.001)	23.9 wpm	+7616%	0.78	(P < 0.001)	0.66	(P < 0.001)
transformative	4.1 wpm	+4489%	0.9	(P < 0.001)	25.7 wpm	+8190%	0.79	(P < 0.001)	0.90	(P < 0.001)
tremendous	15.1 wpm	+1961%	0.87	(P < 0.001)	19.9 wpm	+511%	0.80	(P < 0.001)	0.76	(P < 0.001)

© 2023 Millar N et al. *JAMA Network Open.*

ultimate	-0.3 wpm	-2%	0.37	(P < 0.01)	-37.3 wpm	-44%	-0.50	(P < 0.001)	-0.17	ns
unanswered	6.8 wpm	+3584%	0.8	(P < 0.001)	8.7 wpm	+136%	0.70	(P < 0.001)	0.77	(P < 0.001)
unique	145.7 wpm	+129%	0.92	(P < 0.001)	150.3 wpm	+62%	0.78	(P < 0.001)	0.75	(P < 0.001)
unmet	23 wpm	+12126%	0.86	(P < 0.001)	67.5 wpm	+8039%	0.86	(P < 0.001)	0.79	(P < 0.001)
unparalleled	2.7 wpm	+761%	0.74	(P < 0.001)	12.5 wpm	+1486%	0.88	(P < 0.001)	0.82	(P < 0.001)
unprecedented	24.6 wpm	+2567%	0.89	(P < 0.001)	52.5 wpm	+1219%	0.90	(P < 0.001)	0.87	(P < 0.001)
urgent	25 wpm	+4314%	0.86	(P < 0.001)	74.3 wpm	+3332%	0.89	(P < 0.001)	0.86	(P < 0.001)
user_friendly	7.1 wpm	+1221%	0.82	(P < 0.001)	12.4 wpm	+591%	0.72	(P < 0.001)	0.65	(P < 0.001)
vast	21.9 wpm	+316%	0.81	(P < 0.001)	29.1 wpm	+332%	0.87	(P < 0.001)	0.77	(P < 0.001)
vibrant	0.6 wpm	+244%	0.64	(P < 0.001)	7.2 wpm	+903%	0.83	(P < 0.001)	0.67	(P < 0.001)
vital	36.7 wpm	+374%	0.77	(P < 0.001)	20.0 wpm	+65%	0.67	(P < 0.001)	0.71	(P < 0.001)

Note: wpm = words-per-million

© 2023 Millar N et al. *JAMA Network Open.*

eFigure. Yearly frequency in PubMed abstracts and in NIH funding application abstracts of hype adjectives in descending order of cross-correlation

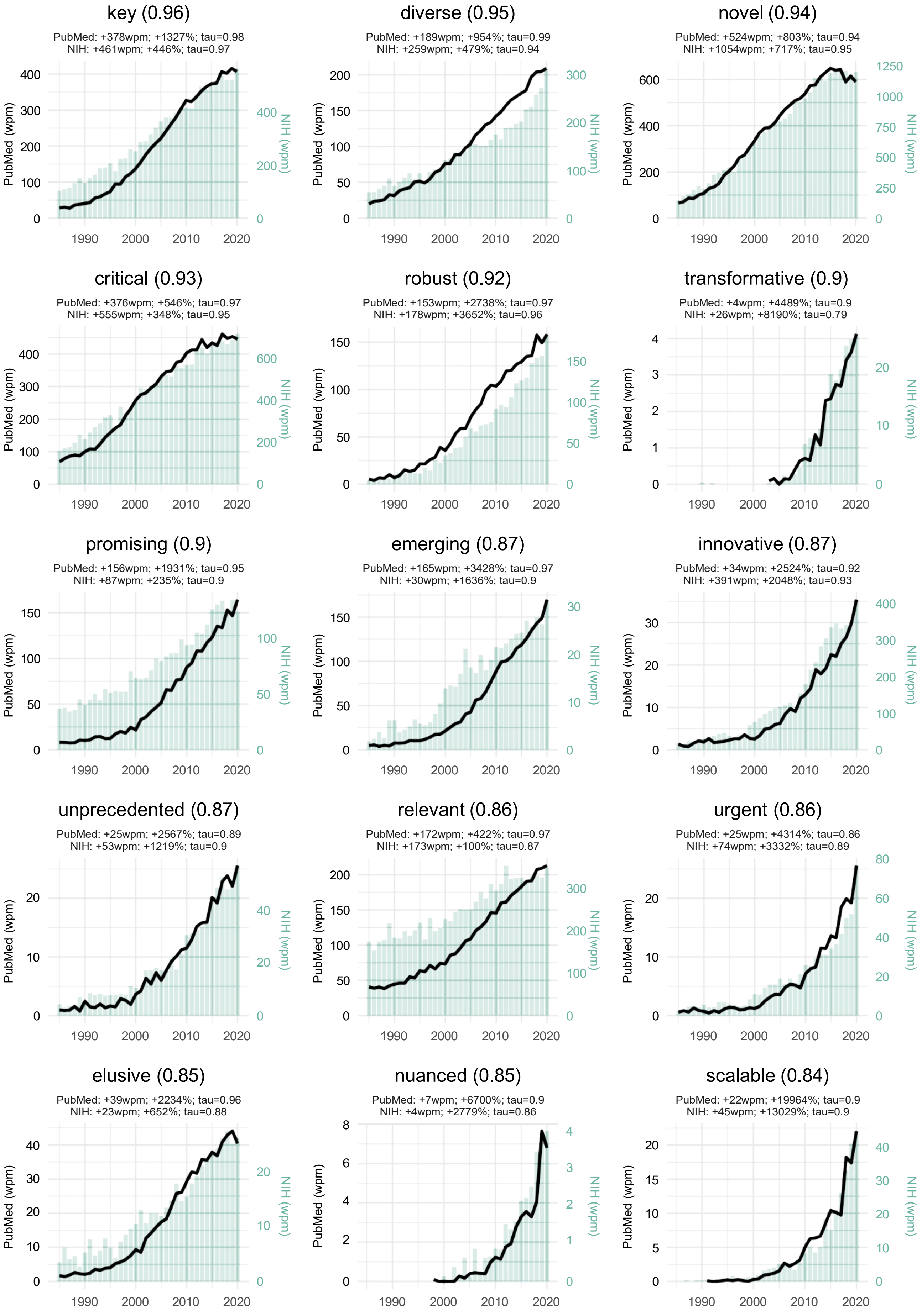

© 2023 Millar N et al. *JAMA Network Open.*

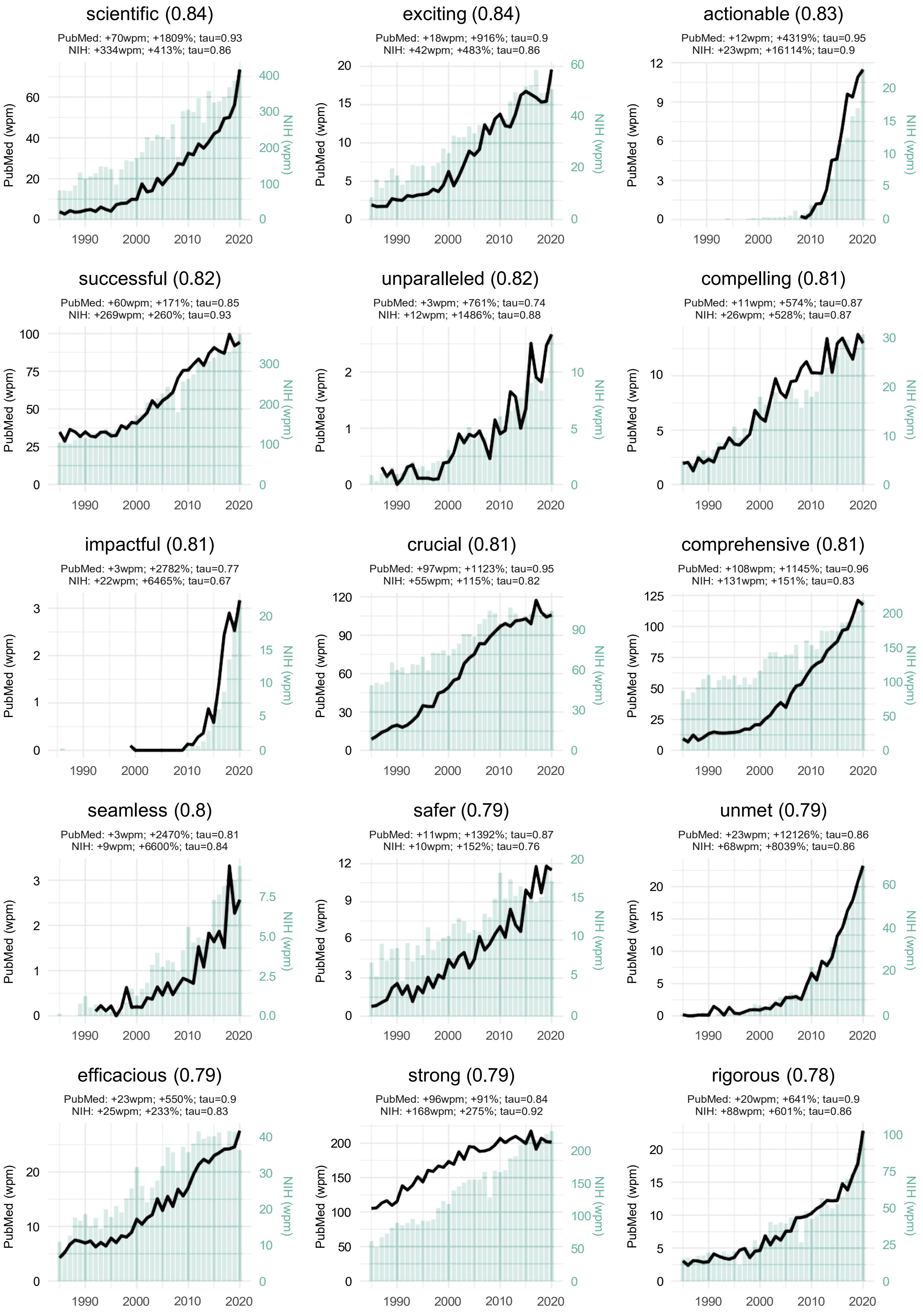

© 2023 Millar N et al. *JAMA Network Open.*

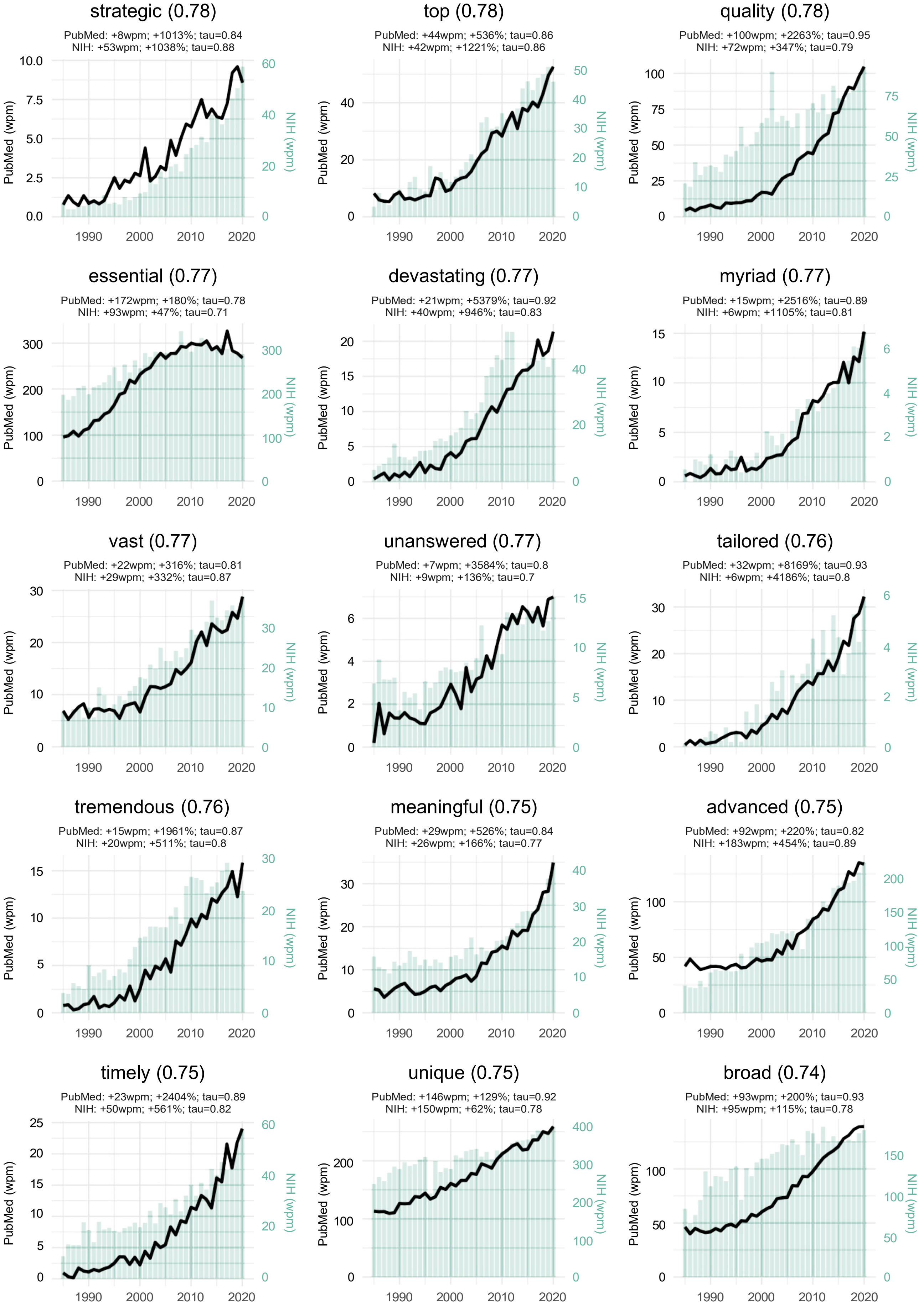

© 2023 Millar N et al. *JAMA Network Open.*

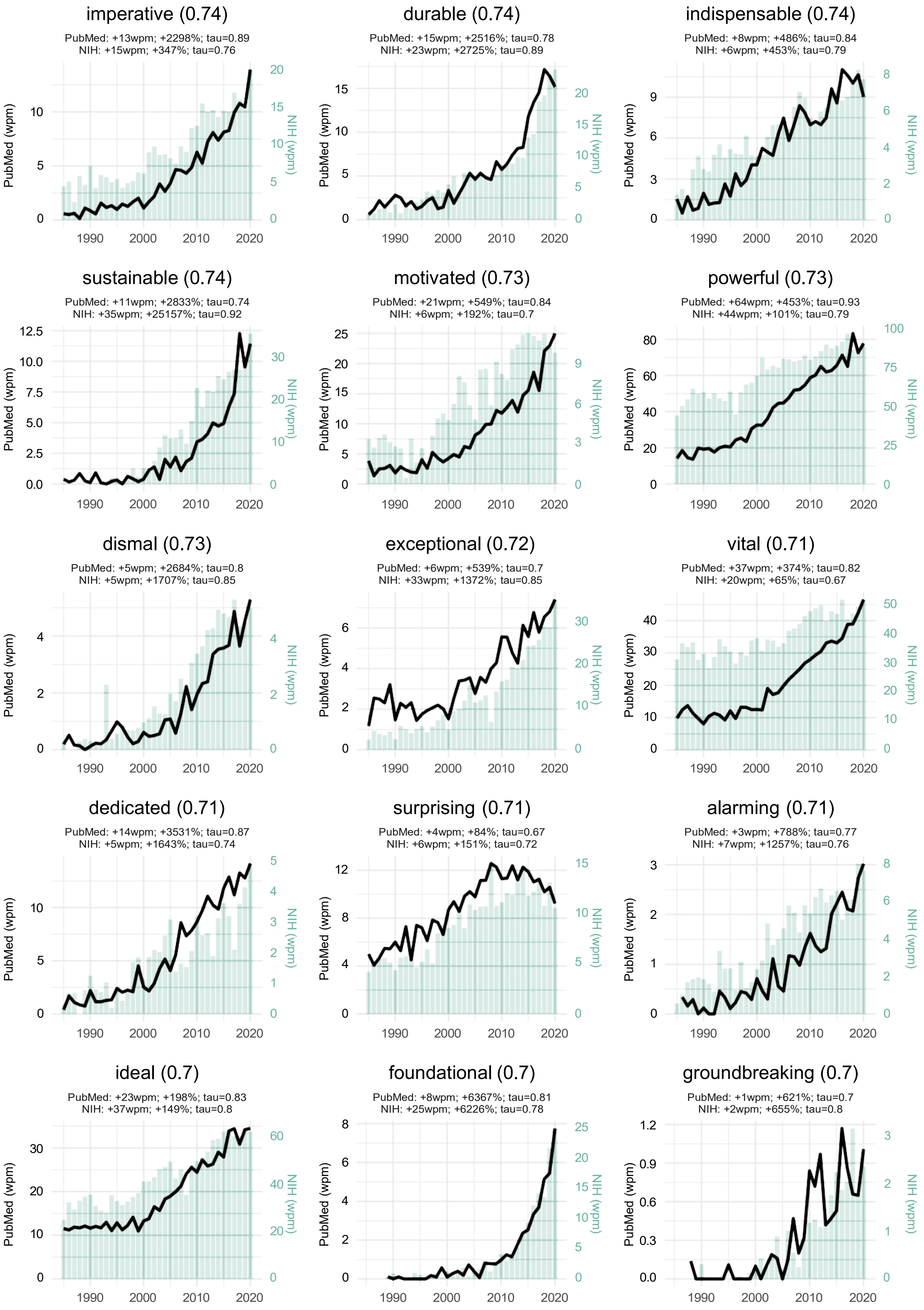

© 2023 Millar N et al. *JAMA Network Open.*

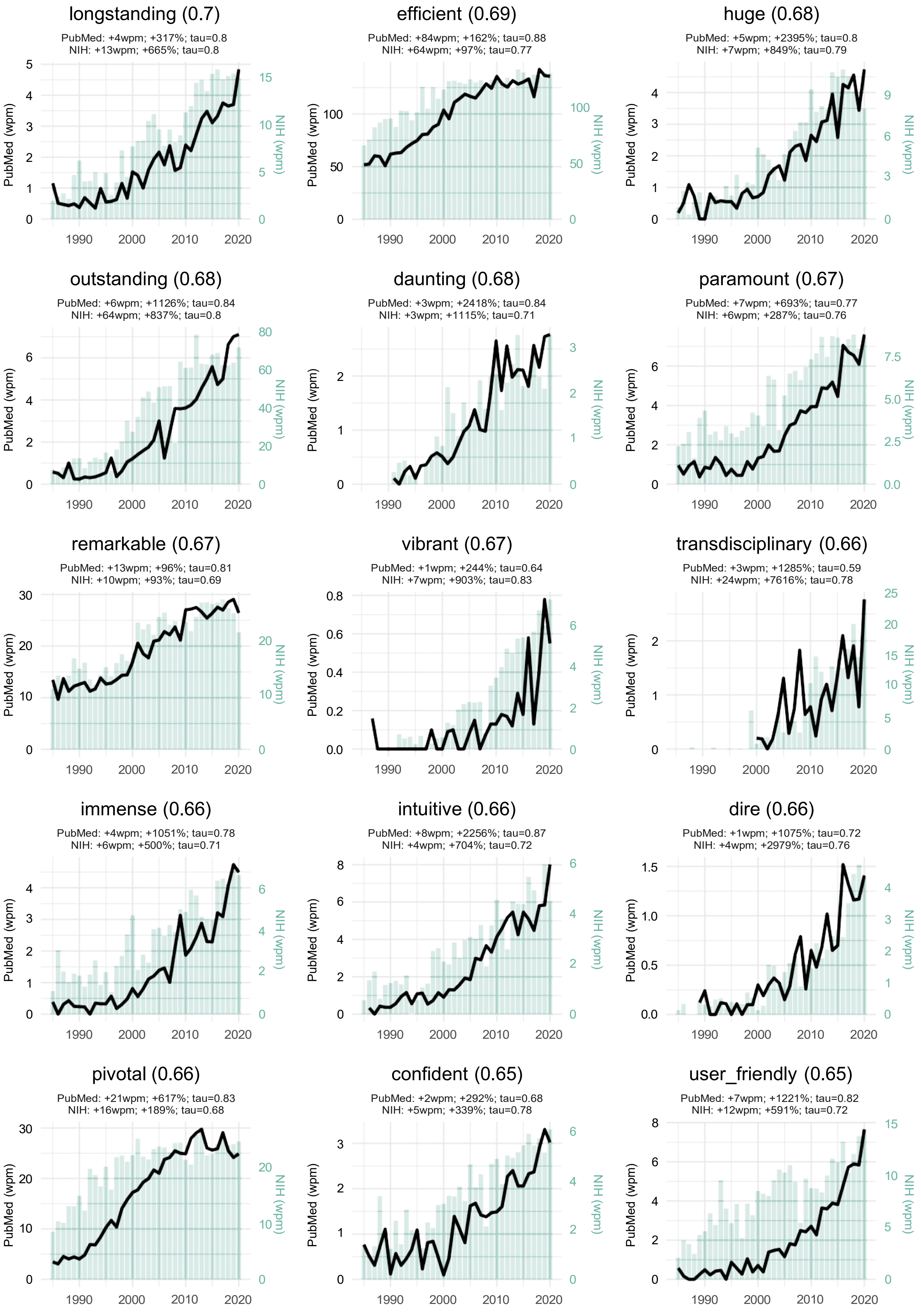

© 2023 Millar N et al. *JAMA Network Open.*

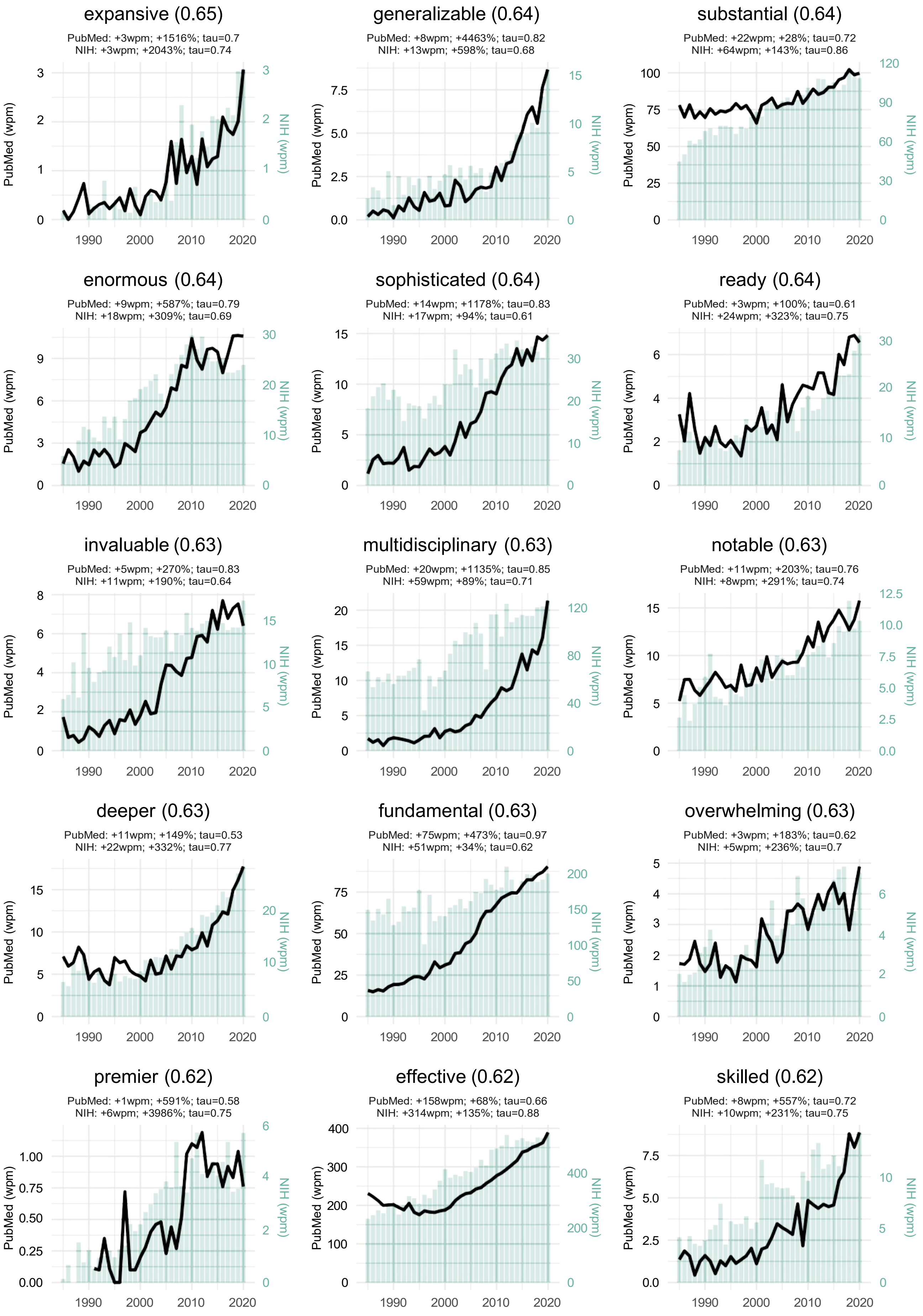

© 2023 Millar N et al. *JAMA Network Open.*

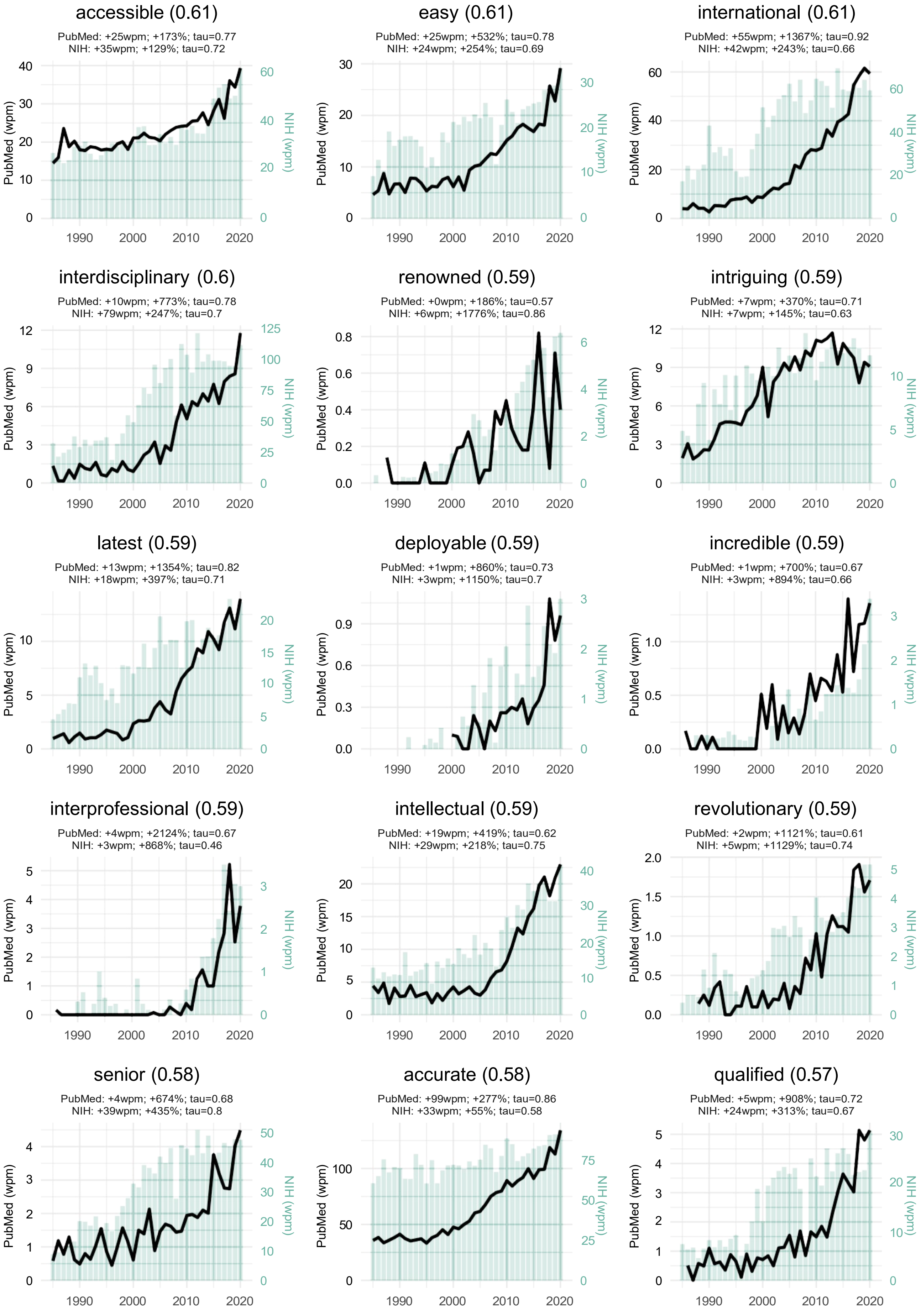

© 2023 Millar N et al. *JAMA Network Open.*

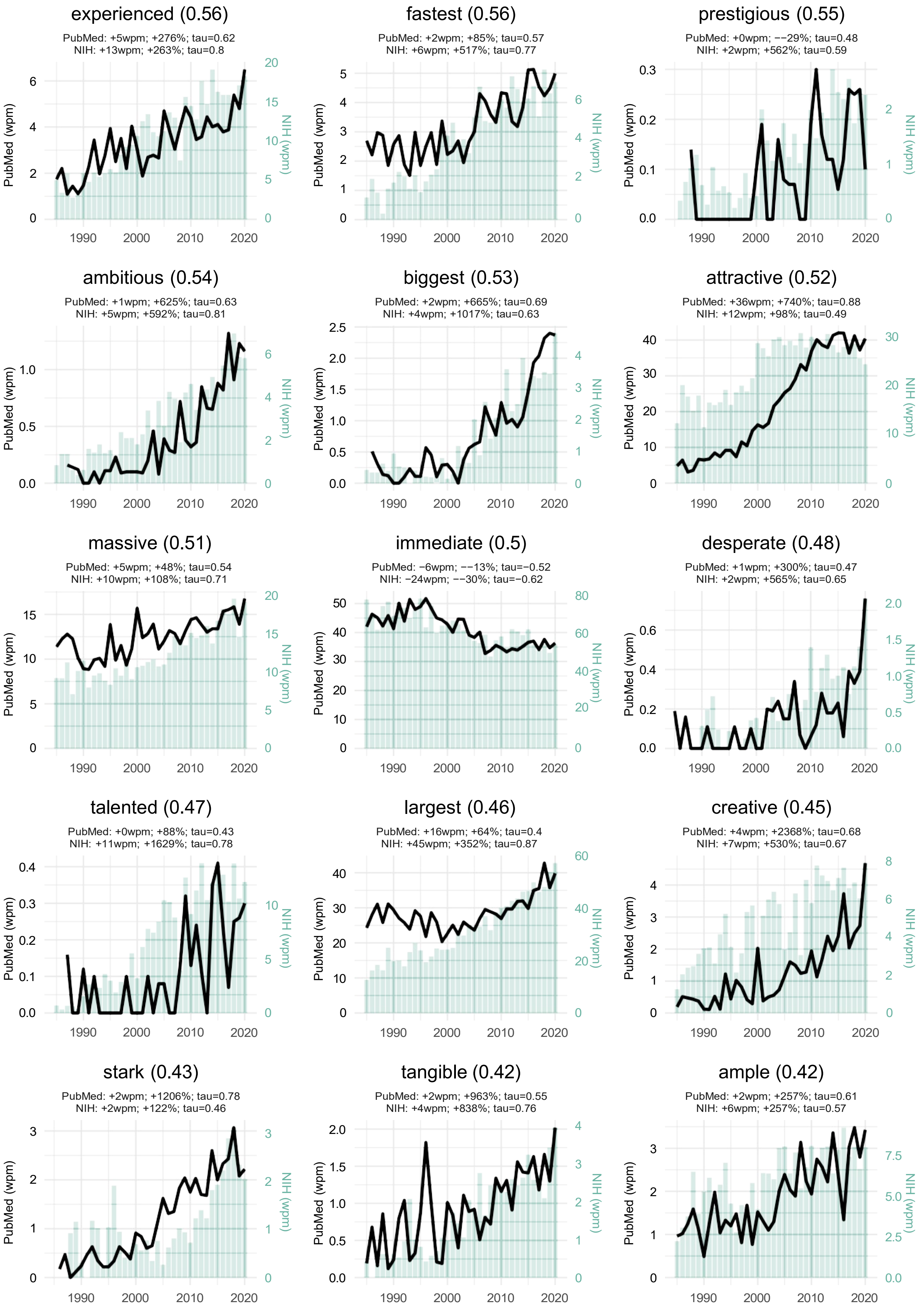

© 2023 Millar N et al. *JAMA Network Open.*

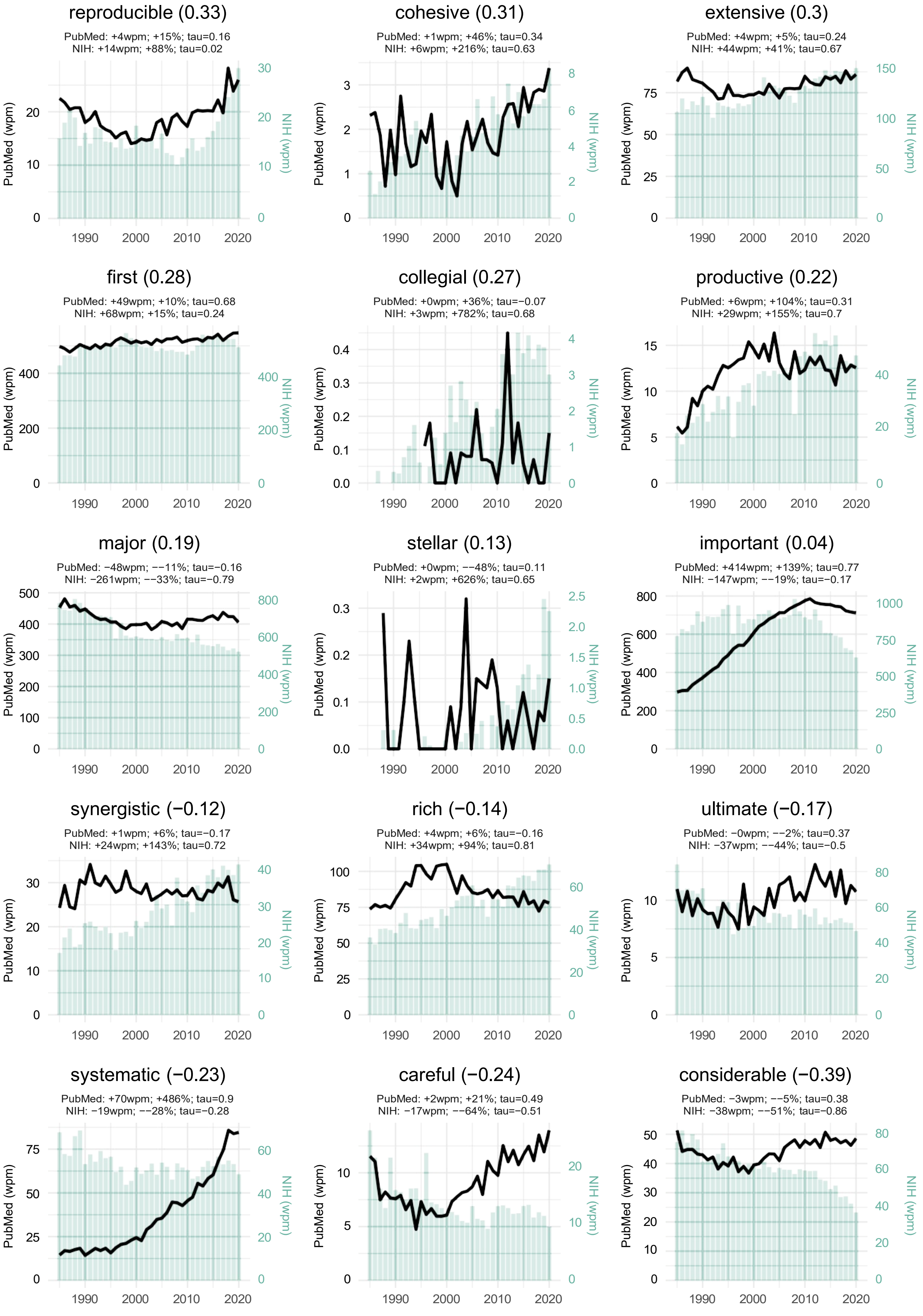

© 2023 Millar N et al. *JAMA Network Open.*

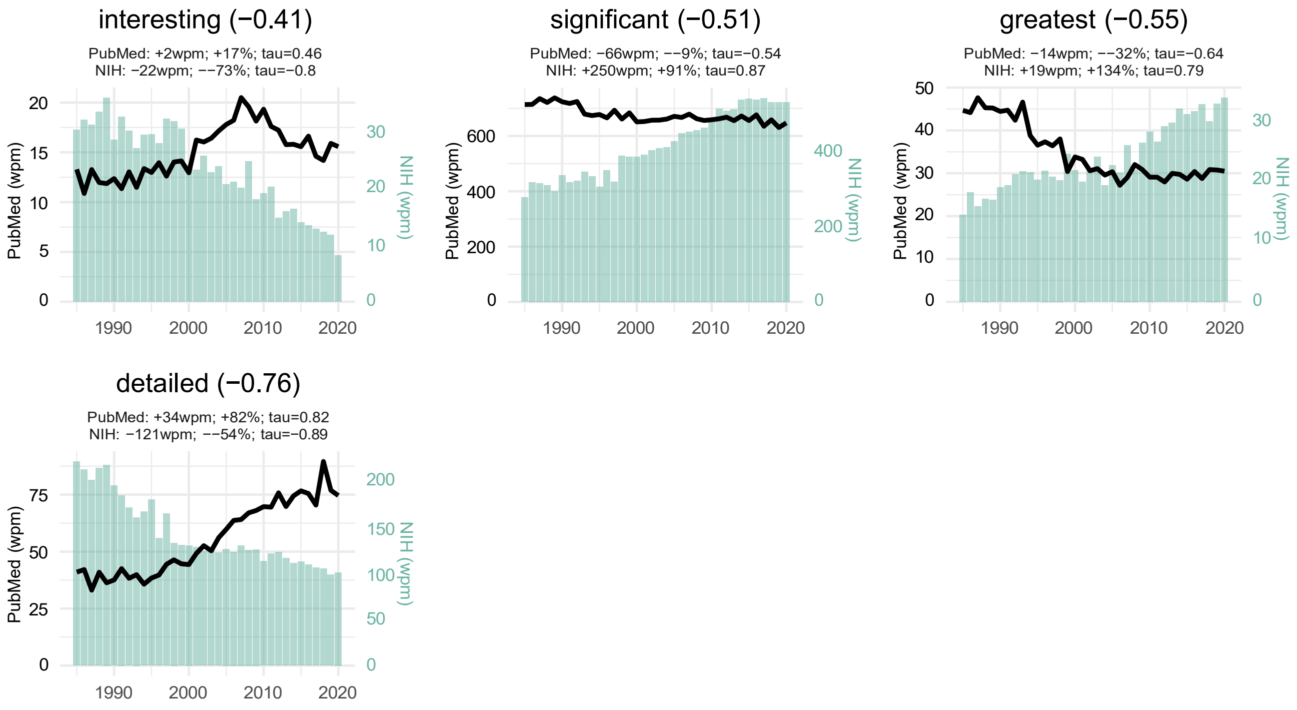

Note: Frequency of each word's use in PubMed abstracts and NIH funding application abstracts is shown (wpm = words-per-million). In the panel label in parentheses the correlation coefficient between word frequency in the two datasets is given, and below that and the absolute change, relative change, and the correlation coefficient in the respective datasets.

© 2023 Millar N et al. *JAMA Network Open.*
