# Supplementary material for: Promotional Language (Hype) in Abstracts of Publications of National Institutes of Health–Funded Research, 1985-2020
Source: JAMA Netw Open. 2023 Dec 21;6(12):e2348706. doi: 10.1001/jamanetworkopen.2023.48706 (PMC10739072; doi:10.1001/jamanetworkopen.2023.48706)
Supplement: Supplement 2. — Data Sharing Statement [file jamanetwopen-e2348706-s002.pdf]

Data Sharing Statement

Millar. Promotional Language (Hype) in Abstracts of Publications of National Institutes of Health–Funded Research, 1985-2020. *JAMA Netw Open*. Published December 21, 2023. doi:10.1001/jamanetworkopen.2023.48706

Data
Data available: Yes
Data types: Other (please specify)
Additional Information: All raw data used in this study is in the public domain and available for download from the NIH Exporter system and from PubMed.
How to access data: https://pubmed.ncbi.nlm.nih.gov/ https://reporter.nih.gov/exporter
When available: With publication

Supporting Documents
Document types: None

Additional Information
Who can access the data: anyone requesting the data
Types of analyses: for any purpose
Mechanisms of data availability: without investigator support
